# Expanded Allelic Diversity of Non‐Classical *HLA* Class I and *MIC* Loci Identified Using Full‐Gene Hybrid Capture–Based Next‐Generation Sequencing

**DOI:** 10.1111/tan.70824

**Published:** 2026-06-28

**Authors:** Itta Krishna Chaaithanya, Pawan Kumar Raghav, Nancy Lee, Carla Wirtz, Denice Kong, Raja Rajalingam

**Affiliations:** ^1^ Immunogenetics and Transplantation Laboratory (ITL), University of California, San Francisco (UCSF) San Francisco California USA; ^2^ Department of Molecular Immunology and Microbiology Indian Council of Medical Research‐National Institute for Research in Reproductive and Child Health (ICMR‐NIRRCH) Mumbai India; ^3^ Academy of Scientific and Innovative Research (AcSIR) Ghaziabad Uttar Pradesh India; ^4^ CareDx West Chester Pennsylvania USA

**Keywords:** full‐gene *HLA* sequencing, *HLA‐E*, *HLA‐F*, *HLA‐G*, *HLA‐H*, hybrid capture–based NGS, *MICA* and *MICB*, non‐classical *HLA* Class I, novel HLA alleles

## Abstract

Non‐classical HLA Class I genes (*HLA‐E*, *‐F*, ‐*G*, ‐*H*) and MHC‐related genes (*MICA*, *MICB*) regulate NK cell function and immune tolerance, yet their allelic diversity remains underexplored. Using capture‐enriched full‐gene sequencing of 943 healthy US transplant donors, we identified 26 novel alleles across *HLA‐E* (2), *HLA‐F* (6), *HLA‐G* (4), *HLA‐H* (5), *MICA* (3), and *MICB* (6). Each novel allele was identified in a single individual and differs from its closest known allele by a single‐nucleotide polymorphism within the coding region. In addition, we confirmed the recently described allele HLA‐G*01:01:33 in 16 unrelated donors. In all cases, it consistently co‐segregated with HLA‐A*34:01:01, HLA‐F*01:01:01 and HLA‐H*02:27, supporting the presence of a conserved haplotype. These findings expand the catalogue of non‐classical *HLA* and *MIC* alleles, highlighting capture‐enriched full‐gene sequencing as a powerful tool for comprehensive immunogenetic profiling.

## 1 Introduction

The HLA system forms the cornerstone of immune recognition and host defence [1, 2]. The human major histocompatibility complex (*MHC*) Class I region contains six functional *HLA* Class I genes [3]. Among these, *HLA‐A*, *HLA‐B* and *HLA‐C* are highly polymorphic and encode classical Class I molecules that are broadly expressed, whereas *HLA‐E*, *HLA‐F* and *HLA‐G* are relatively conserved and encode non‐classical Class I molecules [4, 5]. *HLA‐H* is a non‐classical HLA Class I pseudogene. The MHC Class I chain–related genes *MICA* and *MICB* are also located within the Class I region. All functional HLA Class I and MIC molecules interact with natural killer (NK) cell receptors, CD8^+^ T cells and innate lymphocytes, thereby contributing to immune tolerance, stress surveillance and modulation of inflammatory responses [6, 7, 8]. Their roles have been increasingly recognised in transplantation, pregnancy, autoimmunity, cancer and infectious diseases. Notably, conserved motifs on certain classical HLA Class I molecules, such as C1, C2 and Bw4, as well as the HLA‐A3 and HLA‐A11 molecules, serve as ligands for NK cell receptors [9]. However, the extent of sequence diversity within non‐classical *HLA* Class I and *MIC* genes across global populations—and the functional implications of such polymorphisms for NK cell biology—remain incompletely understood.

As of 2025, these loci account for only 3.7% of all alleles in the IPD‐IMGT/HLA Database, with substantially fewer variants reported for *HLA‐E*, *HLA‐F*, *HLA‐G*, *MICA* and *MICB* compared with classical Class I genes [10]. Earlier genotyping methods primarily targeted exons encoding peptide‐binding domains, overlooking the α3 domain as well as regulatory and intronic regions, where subtle polymorphisms may profoundly affect receptor binding and gene expression [11].

The advent of high‐resolution next‐generation sequencing (NGS) has revolutionised *HLA* genotyping by enabling full‐gene coverage from the 5′ untranslated region (UTR) to the 3′ UTR, facilitating detection of variants across exons, introns and non‐coding regulatory regions [12]. Hybrid capture–based NGS platforms now allow accurate phasing and identification of rare or low‐frequency alleles that were previously undetectable [13].

In this study, we analysed high‐resolution sequencing data from 943 healthy US kidney transplant donors to identify and characterise novel alleles across all non‐classical *HLA* Class I and *MIC* loci. By leveraging full‐gene coverage and stringent validation criteria, we report a comprehensive catalogue of newly identified *HLA‐E*, *HLA‐F*, *HLA‐G*, *HLA‐H*, *MICA* and *MICB* alleles, revealing previously unrecognised genetic diversity and its potential immunological significance.

## 2 Materials and Methods

### 2.1 Study Population and DNA Samples

Between February 2022 and August 2024, a total of 943 high‐resolution hybrid capture–based NGS HLA typing datasets were retrospectively retrieved from unrelated, healthy kidney and haematopoietic stem cell (HSC) transplant donors recorded in the Laboratory Information System of the Immunogenetics and Transplantation Laboratory (ITL) at the University of California, San Francisco (UCSF). All samples were fully de‐identified prior to analysis. The study was approved by the UCSF Institutional Review Board (IRB) and conducted in accordance with institutional and international ethical guidelines for research involving human biological materials and genetic data.

### 2.2 High‐Resolution HLA Typing Using Hybrid Capture NGS

Genomic DNA (gDNA) was isolated from peripheral blood using the Qiagen EZ1 and EZ2 DNA Blood Extraction Kits (Valencia, CA, USA). Only samples meeting strict quality criteria—DNA concentration ≥ 30 ng/μL and an A260/280 ratio between 1.7 and 2.1—were selected for sequencing. High‐resolution genotyping was performed using the CareDx AlloSeq Tx17 hybrid capture–based NGS workflow (CareDx, Brisbane, CA, USA), which provides full‐gene coverage for both classical (*HLA‐A*, *‐B*, *‐C*) and non‐classical *HLA* genes (*HLA‐E*, *‐F*, *‐G*, *‐H*) as well as Class II and *MIC* loci (*MICA*, *MICB*).

Briefly, a minimum of 100 ng of gDNA was first subjected to tagmentation using bead‐linked transposomes (BLTs), which simultaneously fragmented the DNA and added adapter sequences. The reaction product was purified to remove excess BLTs, followed by a limited‐cycle PCR amplification to incorporate dual indices (i7 and i5) and universal adapter sequences (P5 and P7) required for sample identification and cluster generation. Indexed libraries were then pooled, size‐selected and purified using magnetic bead–based cleanup. Target enrichment was subsequently performed using the biotinylated HLA‐specific capture probes provided within the AlloSeq Tx17 hybrid‐capture kit. Captured fragments were isolated using streptavidin‐coated magnetic beads, in accordance with the manufacturer's protocol.

Following enrichment and clean‐up, libraries were quantified using the Qubit dsDNA BR assay (Life Technologies, Grand Island, NY, USA), normalised to 4 nM, denatured with 0.2 N sodium hydroxide (G‐Biosciences, St. Louis, MO, USA) and diluted to 12 pM for optimal cluster density. Sequencing was performed on the Illumina MiSeq platform (Illumina, San Diego, CA, USA) using the MiSeq Reagent Kit v2 (300‐cycle). The MiSeq system uses reversible terminator chemistry to detect nucleotide incorporation in real time.

Quality control (QC) metrics required sequencing data to meet the following predefined thresholds: a minimum read depth of ≥ 500×, allele balance ≥ 30% and ≥ 98% gene coverage. In addition, base quality and mapping quality thresholds of Q30 and MQ40, respectively, were applied in accordance with the CareDx AlloSeq Tx17 workflow specifications.

Novel single‐nucleotide polymorphisms (SNPs) were phased using the Assign haplotype assembly algorithm. Phasing accuracy was further evaluated through manual review of read alignments in the variant regions and confirmed by repeat library preparation and resequencing of the corresponding samples. Only variants that met all QC criteria and demonstrated clear, unambiguous phasing were classified as novel.

### 2.3 Sequence Alignment and Allele Assignment

Raw sequencing reads (FASTQ files) were analysed using CareDx Assign software, which aligns reads to reference sequences from the IPD‐IMGT/HLA Database (Version 3.57; July 2024). A consensus sequence was generated for each locus per sample. The analysis pipeline identified nucleotide mismatches, insertions/deletions (indels), phasing inconsistencies and low‐frequency variants across exonic, intronic and UTRs. Allele assignments were made based on full‐gene or exon‐complete alignments, as specified by the manufacturer.

### 2.4 Detection and Validation of Novel Alleles

The hybrid capture platform provides full‐gene sequencing coverage for each locus, including the 5′ UTR, all exons, all introns and the 3′ UTR. Sequences that did not match any known alleles in the IPD‐IMGT/HLA Database were automatically flagged as candidate novel alleles. Each candidate novel allele underwent a rigorous, multi‐step validation process: (1) re‐examination of raw sequencing reads to exclude sequencing artefacts, mapping errors or index misassignments; (2) manual review of variant positions, zygosity and phasing accuracy; (3) retyping of DNA samples containing candidate alleles for confirmation.

Accurate assignment of fourth‐field variation remains technically challenging in routine short‐read NGS workflows. This limitation reflects several factors: variable or incomplete coverage of intronic and UTR regions; incomplete or poorly characterised reference sequences for many alleles; limited ability of short‐read platforms to establish long‐range phase across extended non‐coding regions; and the higher susceptibility of low‐frequency intronic variants to sequencing and phasing errors. In addition, validation studies for most clinical NGS assays support reliable performance through the third‐field level, whereas fourth‐field resolution is reduced, allele‐dependent and often exceeds established performance specifications.

For these reasons, allele assignments were reported at the highest validated resolution supported by sequencing depth, phasing confidence and assay performance characteristics. Only variants with unambiguous sequencing support and validated phasing were retained. A total of five sequences were excluded from the analysis due to unresolved phasing ambiguity or incomplete representation of heterozygous alleles. Confirmed novel alleles were classified by mutation type (missense, synonymous or non‐coding) and by genomic location (exon, intron or UTR).

### 2.5 Database Submission and Nomenclature Assignment

All validated novel alleles were submitted to GenBank (NCBI) and subsequently to the IPD‐IMGT/HLA Database in accordance with official submission procedures. Formal allele names were assigned by the World Health Organization (WHO) Nomenclature Committee for Factors of the HLA System.

## 3 Results

Full‐length sequencing of 943 unrelated individuals from Northern California identified 26 novel alleles across the non‐classical HLA and MIC loci, including *HLA‐E* (*n* = 2), *HLA‐F* (*n* = 6), *HLA‐G* (*n* = 4), *HLA‐H* (*n* = 5), *MICA* (*n* = 3) and *MICB* (*n* = 6) (Table 1). Each novel allele was identified in a single individual and differs from its closest known allele by a SNP within the coding region. For individuals carrying these novel alleles, the corresponding *HLA‐A*, *‐B*, *‐C*, *‐DRB1*, *‐DQB1*, *‐E*, *‐F*, *‐G*, *‐H*, *MICA* and *MICB* alleles are provided in Table S1.

**TABLE 1:** Novel non‐classical *HLA* Class I and *MIC* alleles identified by full‐length sequencing.

Novel allele	Closest allele	Changes in the novel allele relative to its closest allele					Sequence derived from	GenBank accession number	IPD‐IMGT/HLA submission ID	Race
		Exon	Codon and nucleotide position	Codon change	Amino acid change	SNP effect				
E*01:01:47	E*01:01:01:01	Exon 4	211.3	GC**G**≫GC**A**	A→A	Synonymous	UCSF‐ITL‐001	PQ585392	HWS10093505	A
E*01:153	E*01:03:02:06	Exon 3	115.1	**C**AG≫**G**AG	Q→E	Non‐synonymous	UCSF‐ITL‐002	PQ585393	HWS10093507	A
F*01:25	F*01:01:01:09	Exon 2	85.2	T**A**C≫T**G**C	Y→C	Non‐synonymous	UCSF‐ITL‐003	PQ585385	HWS10093515	U
F*01:26	F*01:03:01:01	Exon 2	20.1	**C**CC≫**T**CC	P→S	Non‐synonymous	UCSF‐ITL‐004	PQ585386	HWS10093517	W
F*01:27	F*01:01:01:08	Exon 3	160.2	C**T**G≫C**C**G	L→P	Non‐synonymous	UCSF‐ITL‐005	PQ585387	HWS10093519	W
F*01:28	F*01:01:01:09	Exon 2	3.3	CA**C**≫CA**A**	H→Q	Non‐synonymous	UCSF‐ITL‐006	PQ585388	HWS10093521	A
F*01:29	F*01:01:01:08	Exon 4	227.2	G**A**C≫G**G**C	D→G	Non‐synonymous	UCSF‐ITL‐007	PQ585389	HWS10093523	W
F*01:01:12	F*01:01:01:09	Exon 3	138.3	AC**C**≫AC**A**	T→T	Synonymous	UCSF‐ITL‐008	PQ585390	HWS10093525	A
G*01:58	G*01:01:03:02	Exon 3	106.1	**G**AC≫**C**AC	D→H	Non‐synonymous	UCSF‐ITL‐009	PQ641881	HWS10093689	W
G*01:59	G*01:01:01:01	Exon 2	29.1	**G**AC≫**T**AC	D→Y	Non‐synonymous	UCSF‐ITL‐010	PQ641884	HWS10093695	W
G*01:60	G*01:06:01:01	Exon 2	85.2	T**A**C≫T**G**C	Y→C	Non‐synonymous	UCSF‐ITL‐011	PQ641885	HWS10093697	A
G*01:61	G*01:01:02:01	Exon 2	69.1	**G**CC≫**C**CC	A→P	Non‐synonymous	UCSF‐ITL‐012	PQ641886	HWS10093699	O
H*01:01:04	H*01:01:01:01	Exon 4	247.3	GA**C**≫GA**T**	—	Pseudogene	UCSF‐ITL‐013	PQ585368	HWS10093605	W
H*02:29	H*02:07:01:01	Exon 4	272.1	**G**TG≫**A**TG	—	Pseudogene	UCSF‐ITL‐014	PQ585367	HWS10093603	A
H*02:30	H*02:07:01:01	Exon 4	262.1	**G**AT≫**A**AT	—	Pseudogene	UCSF‐ITL‐015	PQ585366	HWS10093611	W
H*02:31	H*02:05:01:01	Exon 2	63.1	**G**AC≫**A**AC	—	Pseudogene	UCSF‐ITL‐016	PQ585373	HWS10093617	W
H*02:33	H*02:05:01:01	Exon 4	251.2	A**C**A≫A**G**A	—	Pseudogene	UCSF‐ITL‐017	PQ585369	HWS10093607	O
MICA*002:13	MICA*002:01	Exon 6	359.3	GG**C**≫GG**T**	G→G	Synonymous	UCSF‐ITL‐018	PQ585375	HWS10093531	U
MICA*002:14	MICA*002:01	Exon 4	199.3	AC**C**≫AC**T**	T→T	Synonymous	UCSF‐ITL‐019	PQ585377	HWS10093535	B
MICA*294	MICA*010:01	Exon 3	143.2	A**G**G≫A**A**G	R→K	Non‐synonymous	UCSF‐ITL‐020	PQ585376	HWS10093533	U
							UCSF‐ITL‐021	PQ585382	HWS10093553	U
MICB*005:16	MICB*005:02	Exon 1	2.3	GC**C**≫GC**T**	A→A	Synonymous	UCSF‐ITL‐022	PQ585379	HWS10093557	U
MICB*056	MICB*005:02	Exon 2	50.2	G**C**A≫G**T**A	A→V	Non‐synonymous	UCSF‐ITL‐023	PQ585381	HWS10093551	W
MICB*057	MICB*005:02	Exon 5	294.2	A**T**G≫A**C**G	M→T	Non‐synonymous	UCSF‐ITL‐006	PQ585378	HWS10093555	A
MICB*058	MICB*005:02	Exon 3	122.1	**C**TG≫**A**TG	L→M	Non‐synonymous	UCSF‐ITL‐024	PQ585383	HWS10093559	W
MICB*059	MICB*005:02	Exon 4	221.1	**G**TA≫**A**TA	V→I	Non‐synonymous	UCSF‐ITL‐025	PQ585384	HWS10093561	W
MICB*060	MICB*002:01	Exon 3	142.1	**G**TC≫**A**TC	V→I	Non‐synonymous	UCSF‐ITL‐026	PQ585380	HWS10093549	W

*Note:* Novel non‐classical *HLA* Class I and *MIC* alleles were identified by full‐length sequencing and are shown relative to their closest previously reported alleles. Although full‐gene sequencing was performed, all confirmed novel alleles differed from known alleles by single‐exonic SNPs (in bold). Nucleotide and amino acid differences are described by exon number, codon and nucleotide position, codon change, resulting amino acid substitution and predicted SNP effect (synonymous or non‐synonymous). Alleles designated as pseudogenes contain nucleotide substitutions that do not encode translated amino acid changes. GenBank accession numbers and IPD‐IMGT/HLA submission IDs are provided for each novel allele. Race is self‐reported ancestry, categorised using the following standardised codes: W = European Origin; A = Asian; B = African Origin; O = Other (American Natives or Alaska Natives, Native Hawaiian, Pacific Islander and Multiracial); and U = Unknown.

### 3.1 Novel *HLA‐E* Alleles

Two novel *HLA‐E* alleles were identified (E*01:01:47 and E*01:153). E*01:01:47 differed from its closest allele by a synonymous substitution in Exon 4, resulting in no amino acid change. In contrast, E*01:153 harboured a non‐synonymous substitution in Exon 3, leading to an amino acid change.

### 3.2 Novel HLA‐F Alleles

Six novel *HLA‐F* alleles were detected (F*01:01:12, F*01:25, F*01:26, F*01:27, F*01:28 and F*01:29). Most nucleotide changes were in Exons 2 and 3, which encode the antigen‐binding domains of the HLA‐F molecule. Five alleles contained non‐synonymous substitutions, resulting in amino acid changes. One allele (F*01:01:12) carried a synonymous substitution in Exon 3.

### 3.3 Novel *HLA‐G* Alleles

Four novel *HLA‐G* alleles (G*01:58, G*01:59, G*01:60 and G*01:61) were identified, with variants located in Exon 2 or 3. All four alleles contain non‐synonymous substitutions that result in amino acid changes. Among the 943 individuals analysed, the recently described HLA‐G*01:01:33 allele was detected in 16 individuals from diverse ethnic backgrounds. Table S2 summarises the associated HLA haplotypic background. In all cases, HLA‐G*01:01:33 was consistently observed together with HLA‐A*34:01:01, HLA‐F*01:01:01 and HLA‐H*02:27, suggesting the presence of a strong and conserved non‐classical HLA haplotype.

### 3.4 Novel HLA‐H Alleles

Five novel *HLA‐H* alleles (H*01:01:04, H*02:29, H*02:30, H*02:31 and H*02:33) were identified. All nucleotide substitutions occurred in Exon 2 or 4, and as this is a pseudogene, the predicted amino acid sequence was not determined.

### 3.5 Novel MICA Alleles

Three novel *MICA* alleles (MICA*002:13, MICA*002:14 and MICA*294) were identified. Two alleles exhibited synonymous substitutions, while MICA*294 contained a non‐synonymous substitution in Exon 3. Variants were observed across Exons 3–6, indicating sequence diversity throughout the *MICA* coding region.

### 3.6 Novel MICB Alleles

Six novel *MICB* alleles (MICB*005:16, MICB*056, MICB*057, MICB*058, MICB*059 and MICB*060) were identified. The majority of *MICB* variants carried non‐synonymous substitutions, distributed across Exons 1–5. One allele (MICB*005:16) displayed a synonymous substitution in Exon 1. These results indicate substantial protein‐coding variation within the *MICB* locus.

## 4 Discussion

In this study, hybrid capture–enriched full‐gene NGS of non‐classical *HLA* Class I and *MIC* loci in a large cohort of unrelated healthy individuals revealed substantial previously unrecognised allelic diversity. We identified 26 novel alleles across *HLA‐E*, *HLA‐F*, *HLA‐G*, *HLA‐H*, *MICA* and *MICB*, underscoring the power of capture‐enriched, full‐gene sequencing approaches to detect rare and low‐frequency variants that are often missed by exon‐focused or partial typing methods. All novel alleles differed from their closest previously reported counterparts by a single nucleotide substitution, primarily within Exons 2–4, consistent with prior studies demonstrating that point mutations remain a key driver of diversification within *HLA* genes [4, 14].

### 4.1 Diversity of Non‐Classical HLA Class I Alleles

The identification of novel *HLA‐E* alleles supports the notion that, despite its relatively limited polymorphism compared with classical *HLA* Class I genes, the allelic diversity of HLA‐E documented in the IPD‐IMGT/HLA Database continues to expand and includes both synonymous and non‐synonymous variation. Given the central role of HLA‐E in regulating innate immune responses through interactions with CD94/NKG2 receptors [15], even single amino acid substitutions may influence peptide presentation or receptor binding, as suggested by previous functional studies [16, 17].

Although *HLA‐F* remains the least characterised among the non‐classical *HLA* Class I genes, growing evidence supports its critical role in maternal immune tolerance and placental development through interactions with uterine NK cells and other immune subsets [18]. The relatively high number of novel *HLA‐F* alleles identified in this cohort is therefore noteworthy. The identification of multiple novel HLA‐F alleles suggests that additional allelic sequence diversity at the HLA‐F locus remains to be documented in current reference databases. These variants may reflect the functional relevance of HLA‐F in reproductive immune modulation [19].

Similarly, the identification of four novel *HLA‐G* alleles further expands the allelic sequence diversity of this immunotolerant molecule. HLA‐G exhibits restricted tissue expression and plays critical roles in immune tolerance, including maternal–foetal tolerance and immune regulation in transplantation and cancer [20, 21]. The predominance of non‐synonymous substitutions among newly identified *HLA‐G* alleles suggests that functional variation at this locus may be more extensive than previously appreciated. Such changes may affect interactions with inhibitory receptors such as LILRB1 and KIR2DL4, with potential consequences for immune tolerance and transplant outcomes [22].

### 4.2 Conserved Haplotypic Linkage of HLA‐G*01:01:33

A particularly notable finding was the detection of recently identified HLA‐G*01:01:33 in 16 unrelated individuals. This allele was consistently observed in combination with HLA‐A*34:01:01, HLA‐F*01:01:01 and HLA‐H*02:27, forming a conserved non‐classical *HLA* haplotype across all carriers. Such a strong linkage across the *HLA‐A* and non‐classical *HLA* Class I region has been described previously and reflects limited recombination within this genomic segment [23]. The repeated detection of this extended haplotype in individuals from different ethnic backgrounds suggests that HLA‐G*01:01:33 likely arose on a stable ancestral background and may represent an underreported haplotype rather than a sporadic variant.

### 4.3 HLA‐H Variation and Pseudogene Stability

HLA‐H is a nonfunctional pseudogene, and five novel HLA‐H alleles were identified in this cohort. Although it does not encode a functional protein, documenting allelic diversity at this locus remains important for accurate haplotype reconstruction and for understanding the evolutionary dynamics of the HLA Class I region.

### 4.4 Novel MIC Alleles and Implications for Immune Recognition

MICA and MICB interact with NKG2D, an activating C‐type lectin‐like immunoreceptor expressed on natural killer cells, γδ T cells and CD8^+^ αβ T cells [24]. Through NKG2D‐mediated cytolytic activation, these ligands contribute to the early immune response against pathogens and tumours [25]. Among the newly identified alleles, several *MICA* variants involved synonymous substitutions, whereas most *MICB* variants contained non‐synonymous changes that altered the encoded protein sequence. This observation aligns with previous findings indicating greater functional diversity within *MICB* compared with *MICA* [26]. Consequently, even single amino acid substitutions may modulate ligand stability, receptor binding and immune recognition.

### 4.5 Homozygous Whole‐Gene Deletions of MICB and HLA‐H

Interestingly, we identified four individuals with apparent homozygous whole‐gene deletions of *MICB* and one individual who appeared completely negative for *HLA‐H*. These findings were reproduced on repeat testing. We did not observe any shared alleles at other HLA loci that were consistently associated with *MICB* negativity. However, given the unexpected nature of complete gene deletions in this genomic region, these findings require confirmation in larger cohorts and through more targeted genomic approaches, such as dedicated structural variant analysis or long‐read sequencing, to exclude technical artefacts and to validate true germline gene absence.

Apparent absence of HLA‐H in these samples is consistent with previously described haplotype‐associated contraction of the HLA‐A subregion in HLA‐A24 (and A23) haplotypes, which can encompass deletion of the HLA‐H locus [27]. Therefore, the HLA‐H negative result likely reflects known haplotypic variation rather than a sequencing artifact.

### 4.6 Broader Implications and Limitations

The predominance of rare, single‐occurrence alleles highlights the depth of unexplored diversity within non‐classical HLA and MIC genes, even in well‐characterised populations. These findings underscore the importance of continuously updating immunogenetic databases, as rare variants may help explain differences in immune responses between individuals.

A limitation of this study is the absence of functional validation for the newly identified variants. Future investigations will be required to determine whether observed non‐synonymous substitutions alter protein expression, stability, or receptor interactions. Additionally, larger and more diverse population studies will be necessary to define the geographic and ethnic distribution of these alleles and to clarify whether alleles such as HLA‐G*01:01:33 represent population‐enriched haplotypes.

## 5 Conclusion

In summary, this study substantially expands the catalogue of non‐classical *HLA* and *MIC* alleles, demonstrating the value of capture‐enriched full‐gene sequencing for comprehensive immunogenetic characterisation. Continued identification and documentation of rare and novel variants will be essential for advancing our understanding of immune diversity and its clinical relevance.

## Author Contributions

I.K.C. and R.R. conceived and designed the study. I.K.C. performed data analysis and validation and drafted the manuscript. N.L. and C.W. contributed to methodology and data acquisition. D.K. and P.K.R. contributed to data analysis and management. R.R. supervised the study, secured funding and critically revised the manuscript. All authors approved the final version and agreed to be accountable for all aspects of the work.

## Funding

This work was supported by ICMR‐DHR International Fellowship for Young Indian Biomedical Scientists.

## Conflicts of Interest

The authors declare no conflicts of interest.

## Supporting information


**Table S1:** Novel non‐classical HLA and MIC alleles identified by full‐gene sequencing.

**Table S2:** Linkage of the novel allele HLA‐G*01:01:33 with HLA‐A*34:01:01, HLA‐F*01:01:01 and HLA‐H*02:27.

## Data Availability

The data that support the findings of this study are available on request from the corresponding author. The data are not publicly available due to privacy or ethical restrictions.

## References

[1] J. Klein and A. Sato , “The HLA System. Second of Two Parts,” New England Journal of Medicine 343, no. 11 (2000): 782–786. 10984567 10.1056/NEJM200009143431106

[2] J. Klein and A. Sato , “The HLA System. First of Two Parts,” New England Journal of Medicine 343, no. 10 (2000): 702–709. 10974135 10.1056/NEJM200009073431006

[3] R. Horton , L. Wilming , V. Rand , et al., “Gene Map of the Extended Human MHC,” Nature Reviews. Genetics 5, no. 12 (2004): 889–899. 10.1038/nrg1489 15573121

[4] J. Robinson , L. A. Guethlein , N. Cereb , et al., “Distinguishing Functional Polymorphism From Random Variation in the Sequences of >10,000 HLA‐A, ‐B and ‐C Alleles,” PLoS Genetics 13, no. 6 (2017): e1006862. 28650991 10.1371/journal.pgen.1006862 PMC5507469

[5] D. J. Barker , R. H. L. Natarajan , M. A. Cooper , et al., “The IPD‐IMGT/HLA Database: Recent Developments in Sequence Submission,” Nucleic Acids Research 54, no. D1 (2026): D1152–D1158. 41251166 10.1093/nar/gkaf1218 PMC12807722

[6] I. K. Chaaithanya and R. Rajalingam , “Emerging Roles of Natural Killer Cell Ligands‐HLA‐E, HLA‐F, HLA‐G, MICA, and MICB‐In In Vitro Fertilization Outcomes,” Frontiers in Genetics 16 (2025): 1661511. 41321564 10.3389/fgene.2025.1661511 PMC12658362

[7] P. Parham , “MHC Class I Molecules and KIRs in Human History, Health and Survival,” Nature Reviews. Immunology 5, no. 3 (2005): 201–214. 10.1038/nri1570 15719024

[8] M. Lopez‐Botet , M. Llano , F. Navarro , and T. Bellon , “NK Cell Recognition of Non‐Classical HLA Class I Molecules,” Seminars in Immunology 12, no. 2 (2000): 109–119. 10764619 10.1006/smim.2000.0213

[9] R. Rajalingam , “Overview of the Killer Cell Immunoglobulin‐Like Receptor System,” Methods in Molecular Biology 882 (2012): 391–414. 22665247 10.1007/978-1-61779-842-9_23

[10] D. J. Barker , G. Maccari , X. Georgiou , et al., “The IPD‐IMGT/HLA Database,” Nucleic Acids Research 51, no. D1 (2023): D1053–D1060. 36350643 10.1093/nar/gkac1011 PMC9825470

[11] H. Erlich , “HLA DNA Typing: Past, Present, and Future,” Tissue Antigens 80, no. 1 (2012): 1–11. 22651253 10.1111/j.1399-0039.2012.01881.x

[12] D. Kong , N. Lee , I. D. Dela Cruz , et al., “Concurrent Typing of Over 4000 Samples by Long‐Range PCR Amplicon‐Based NGS and rSSO Revealed the Need to Verify NGS Typing for HLA Allelic Dropouts,” Human Immunology 82, no. 8 (2021): 581–587. 33980471 10.1016/j.humimm.2021.04.008

[13] P. J. Norman , J. A. Hollenbach , N. Nemat‐Gorgani , et al., “Defining KIR and HLA Class I Genotypes at Highest Resolution via High‐Throughput Sequencing,” American Journal of Human Genetics 99, no. 2 (2016): 375–391. 27486779 10.1016/j.ajhg.2016.06.023 PMC4974113

[14] A. M. Little and P. Parham , “Polymorphism and Evolution of HLA Class I and II Genes and Molecules,” Reviews in Immunogenetics 1, no. 1 (1999): 105–123. 11256568

[15] S. Lazetic , C. Chang , J. P. Houchins , L. L. Lanier , and J. H. Phillips , “Human Natural Killer Cell Receptors Involved in MHC Class I Recognition Are Disulfide‐Linked Heterodimers of CD94 and NKG2 Subunits,” Journal of Immunology 157, no. 11 (1996): 4741–4745. 8943374

[16] V. Braud , E. Y. Jones , and A. McMichael , “The Human Major Histocompatibility Complex Class Ib Molecule HLA‐E Binds Signal Sequence‐Derived Peptides With Primary Anchor Residues at Positions 2 and 9,” European Journal of Immunology 27, no. 5 (1997): 1164–1169. 9174606 10.1002/eji.1830270517

[17] G. M. Gillespie , M. N. Quastel , and A. J. McMichael , “HLA‐E: Immune Receptor Functional Mechanisms Revealed by Structural Studies,” Immunological Reviews 329, no. 1 (2025): e13434. 39753525 10.1111/imr.13434 PMC11698700

[18] C. E. Dunk , M. Bucher , J. Zhang , et al., “Human Leukocyte Antigen HLA‐C, HLA‐G, HLA‐F, and HLA‐E Placental Profiles Are Altered in Early Severe Preeclampsia and Preterm Birth With Chorioamnionitis,” American Journal of Obstetrics and Gynecology 227, no. 4 (2022): 641.e1–641.e13. 10.1016/j.ajog.2022.07.021 35863458

[19] H. I. Moller , C. M. Christiansen , F. R. Klok , et al., “Investigations of HLA‐F and HLA‐G 3'UTR Polymorphisms in Preeclampsia and Fetal Growth Restriction Indicate a Possible Role of HLA‐F‐HLA‐G Haplotypes and Diplotypes,” HLA 106, no. 1 (2025): e70293. 40603242 10.1111/tan.70293 PMC12221863

[20] N. Rouas‐Freiss , R. M. Goncalves , C. Menier , J. Dausset , and E. D. Carosella , “Direct Evidence to Support the Role of HLA‐G in Protecting the Fetus From Maternal Uterine Natural Killer Cytolysis,” Proceedings of the National Academy of Sciences of the United States of America 94, no. 21 (1997): 11520–11525. 9326642 10.1073/pnas.94.21.11520 PMC23525

[21] P. Contini , G. Murdaca , F. Puppo , and S. Negrini , “HLA‐G Expressing Immune Cells in Immune Mediated Diseases,” Frontiers in Immunology 11 (2020): 1613. 32983083 10.3389/fimmu.2020.01613 PMC7484697

[22] I. Nowak , A. Malinowski , E. Barcz , et al., “Possible Role of HLA‐G, LILRB1 and KIR2DL4 Gene Polymorphisms in Spontaneous Miscarriage,” Archivum Immunologiae et Therapiae Experimentalis (Warsz) 64, no. 6 (2016): 505–514. 10.1007/s00005-016-0389-7 PMC5085992 26973020

[23] F. Carlini , V. Ferreira , S. Buhler , et al., “Association of HLA‐A and Non‐Classical HLA Class I Alleles,” PLoS One 11, no. 10 (2016): e0163570. 27701438 10.1371/journal.pone.0163570 PMC5049754

[24] D. H. Raulet , “Roles of the NKG2D Immunoreceptor and Its Ligands,” Nature Reviews. Immunology 3, no. 10 (2003): 781–790. 10.1038/nri1199 14523385

[25] M. B. Lodoen and L. L. Lanier , “Natural Killer Cells as an Initial Defense Against Pathogens,” Current Opinion in Immunology 18, no. 4 (2006): 391–398. 16765573 10.1016/j.coi.2006.05.002 PMC7127478

[26] S. Bahram , “MIC Genes: From Genetics to Biology,” Advances in Immunology 76 (2000): 1–60. 11079097 10.1016/s0065-2776(01)76018-x

[27] C. P. Venditti and M. J. Chorney , “Class I Gene Contraction Within the HLA‐A Subregion of the Human MHC,” Genomics 14, no. 4 (1992): 1003–1009. 1362179 10.1016/s0888-7543(05)80123-5

